# Supplementary material for: Prevalence and associated factors of depression among adult refugees in East Africa: A protocol for systematic review and meta-analysis
Source: PLoS One. 2025 Feb 11;20(2):e0318904. doi: 10.1371/journal.pone.0318904 (PMC11813089; doi:10.1371/journal.pone.0318904)
Supplement: S1 File — (DOCX) [file pone.0318904.s001.docx]

**S1**: PubMed search strategy for systematic review and meta-analysis protocol on prevalence and associated factors of depression among adult refugees in East Africa

| Search Number | Search Detail |
| --- | --- |
| #1 | “**Prevalences”[MeSH Terms]** |
| #2 | **(((((((Prevalence[Title/Abstract]) OR (Prevalences[Title/Abstract])) OR (Period Prevalence[Title/Abstract])) OR (Period Prevalences[Title/Abstract])) OR (Prevalence, Period[Title/Abstract])) OR (Point Prevalence[Title/Abstract])) OR (Point Prevalences[Title/Abstract])) OR (Prevalence, Point[Title/Abstract])** |
| #3 | **“Depression”[MeSH Terms]** |
| #4 | **((((Depressive Symptoms[Title/Abstract]) OR (Depressive Symptom[Title/Abstract])) OR (Symptom, Depressive[Title/Abstract])) OR (Emotional Depression[Title/Abstract])) OR (Depression, Emotional[Title/Abstract])** |
| #5 | **“Risk Factors”[MeSH Terms]** |
| #6 | **((((((((((((((((((Risk Factors[Title/Abstract]) OR (Factor, Risk[Title/Abstract])) OR (Risk Factor[Title/Abstract])) OR (Associated factors[Title/Abstract])) OR (Determinant factors[Title/Abstract])) OR (contributing factors[Title/Abstract])) OR (Social Risk Factors[Title/Abstract])) OR (Personal Factors[Title/Abstract])) OR (Environmental Factors[Title/Abstract])) OR (Factor, Social Risk[Title/Abstract])) OR (Factors, Social Risk[Title/Abstract])) OR (Risk Factor, Social[Title/Abstract])) OR (Risk Factors, Social[Title/Abstract])) OR (Social Risk Factor[Title/Abstract])) OR (Health Correlates[Title/Abstract])) OR (Correlates, Health[Title/Abstract])) OR (Population at Risk[Title/Abstract])) OR (Populations at Risk[Title/Abstract])) OR (Risk Scores[Title/Abstract])) OR (Risk Score[Title/Abstract])) OR (Score, Risk[Title/Abstract])) OR (Risk Factor Scores[Title/Abstract])) OR (Risk Factor Score[Title/Abstract])) OR (Score, Risk Factor[Title/Abstract]** |
| #7 | "Adult"[MeSH Terms] |
| #8 | "Adults"[Title/Abstract] |
| #9 | **“Refugees”[MeSH Terms]** |
| #10 | **(((((((((((((((((((((Refugee[Title/Abstract]) OR (Political Asylum Seekers[Title/Abstract])) OR (Asylum Seeker, Political[Title/Abstract])) OR (Asylum Seekers, Political[Title/Abstract])) OR (Political Asylum Seeker[Title/Abstract])) OR (Seekers, Political Asylum[Title/Abstract])) OR (Political Refugees[Title/Abstract])) OR (Political Refugee[Title/Abstract])) OR (Refugee, Political[Title/Abstract])) OR (Refugees, Political[Title/Abstract])) OR (Asylum Seekers[Title/Abstract])) OR (Asylum Seeker[Title/Abstract])) OR (Seeker, Asylum[Title/Abstract])) OR (Seekers, Asylum[Title/Abstract])) OR (Displaced Persons[Title/Abstract])) OR (Displaced Person[Title/Abstract])) OR (Person, Displaced[Title/Abstract])) OR (Persons, Displaced[Title/Abstract])) OR (Internally Displaced Persons[Title/Abstract])) OR(Displaced Person, Internally[Title/Abstract])) OR (Displaced Persons, Internally[Title/Abstract])) OR (Internally Displaced Person[Title/Abstract])** |
| #11 | **(((((((((((((Africa, Eastern[MeSH Terms]) OR (Burundi[MeSH Terms])) OR (Comoros[MeSH Terms])) OR (Djibouti[MeSH Terms])) OR (Eritrea[MeSH Terms])) OR (Ethiopia[MeSH Terms])) OR (Kenya[MeSH Terms])) OR (Madagascar[MeSH Terms])) OR (Seychelles[MeSH Terms])) OR (Somalia[MeSH Terms])) OR (South Sudan[MeSH Terms])) OR (Sudan[MeSH Terms])) OR (Tanzania[MeSH Terms])) OR (Uganda[MeSH Terms])** |
| #12 | **(((((((((((((((((East Africa[Title/Abstract]) OR (Eastern Africa[Title/Abstract])) OR (Republic of Burundi[Title/Abstract])) OR (Urundi[Title/Abstract])) OR (Iles Comores[Title/Abstract])) OR (Comoro Islands[Title/Abstract])) OR (Mayotte[Title/Abstract])) OR (Somaliland, French[Title/Abstract])) OR (Republic of Djibouti[Title/Abstract])) OR (French Somaliland[Title/Abstract])) OR (Federal Democratic Republic of Ethiopia[Title/Abstract])) OR (Republic of Kenya[Title/Abstract])) OR (Malagasy Republic[Title/Abstract])) OR (Republic of the Sudan[Title/Abstract])) OR (United Republic of Tanzania[Title/Abstract])) OR (Zanzibar[Title/Abstract])) OR (Tanganyika[Title/Abstract])) OR (Republic of Uganda[Title/Abstract])** |
| #13 | **(#1) OR (#2)** |
| #14 | **(#3) OR (#4)** |
| #15 | **(#5) OR (#6)** |
| #16 | **(#7) OR (#8)** |
| #17 | **(#9) OR (#10)** |
| #18 | **(#11) OR (#12)** |
| #19 | #13 AND #14 AND #15 AND #16 AND #17 AND #18 |

**Google Scholar results**

Depression “Depressive Symptoms“ |”Depressive Symptom" |"Symptom, Depressive" |"Emotional Depression" |"Depression, Emotional" “Refugees” |“Refugee” |"Political Asylum Seekers" |"Asylum Seeker, Political" |"Asylum Seekers, Political" |"Political Asylum Seeker" |"Seekers, Political Asylum" |"Political Refugees" |"Political Refugee" |"Refugee, Political" |"Refugees, Political" |"Asylum Seekers" |"Asylum Seeker" |"Seeker, Asylum" |"Seekers, Asylum" | "Displaced Persons" |"Displaced Person" |"Person, Displaced" |"Persons, Displaced" |"Internally Displaced Persons" |"Displaced Person, Internally" |"Displaced Persons, Internally " |"Internally Displaced Person" "Africa, Eastern" |"East Africa " |"Eastern Africa " |"Burundi" |"Republic of Burundi" |Urundi |"Comoros" |"Comores" |"Comoro Islands" |"Mayotte" |" Djibouti" |"Somaliland, French" |"Republic of Djibouti" |"French Somaliland" |"Eritrea" |"Ethiopia" |"Federal Democratic Republic of Ethiopia" |"Kenya" |"Republic of Kenya" |"Madagascar" |"Malagasy Republic" |"Seychelles" |"Somalia" |"South Sudan" |"Sudan" | "Republic of the Sudan" |"Tanzania" |"United Republic of Tanzania" |"Zanzibar" |"Tanganyika" | "Uganda" |"Republic of Uganda"

**CINAHAL Searching strategy results**

((TitleCombined:(Depression)) OR (TitleCombined:("Depressive Symptom*")) OR (TitleCombined:("Emotional Depression"))) AND ((TitleCombined:(Refugee*)) OR (TitleCombined:("Displaced Person*”)) OR (TitleCombined:("Internally Displaced Person*"))) AND ((TitleCombined:("East* Africa ")) OR (TitleCombined:(Burundi)) OR (TitleCombined:("Republic of Burundi”)) OR (TitleCombined:(Urundi)) OR (TitleCombined:(Comoros)) OR (TitleCombined:(Comores)) OR (TitleCombined:("Comoro Islands")) OR (TitleCombined:(Mayotte)) OR (TitleCombined:(Djibouti)) OR (TitleCombined:("Republic of Djibouti")) OR (TitleCombined:("French Somaliland")) OR (TitleCombined:(Eritrea)) OR (TitleCombined:(Ethiopia)) OR (TitleCombined:("Federal Democratic Republic of Ethiopia")) OR (TitleCombined:( Kenya)) OR (TitleCombined:(“Republic of Kenya”)) OR (TitleCombined:(Madagascar)) OR (TitleCombined:(“Malagasy Republic”)) OR (TitleCombined:( Seychelles)) OR (TitleCombined:( Somalia)) OR (TitleCombined:( “South Sudan”)) OR (TitleCombined:( Sudan)) OR (TitleCombined:(“Republic of the Sudan”)) OR (TitleCombined:(Tanzania)) OR (TitleCombined:( “United Republic of Tanzania”)) OR (TitleCombined:( Zanzibar)) OR (TitleCombined:(Tanganyika)) OR (TitleCombined:(Uganda)) OR (TitleCombined:(“Republic of Uganda”)))

**AJOL Search Strategy Code**

((refugees OR "asylum seekers" OR "displaced persons" OR migrants) AND (depression OR "mental health" OR PTSD OR "psychological distress" OR trauma) AND ("East Africa" OR Ethiopia OR Uganda OR Kenya OR Tanzania OR Somalia OR Rwanda OR Burundi OR "South Sudan" OR Sudan OR Eritrea OR Djibouti) AND (prevalence OR "associated factors" OR determinants OR correlates))
