# Supplementary material for: Prevalence and associated factors of depression among adult refugees in East Africa: A protocol for systematic review and meta-analysis
Source: PLoS One. 2025 Feb 11;20(2):e0318904. doi: 10.1371/journal.pone.0318904 (PMC11813089; doi:10.1371/journal.pone.0318904)
Supplement: Attachment — Submitted filename: Comments on protocol on depression among adult refuges.docx [file pone.0318904.s003.docx]

Title: **Prevalence and associated factors of depression among adult refugees in East Africa: A protocol for systematic review and meta-analysis.**

I would like to thank you the editor to give me the opportunity to review this interested protocol which focused on the unusual/refugee settings. I hope the review protocol will help to design strategies for the improvement of the health of refugees particularly in the settings with natural and man-made catastrophes. Here below are some of my comments.

**Abstract**

Line 23--- I think web of sciences needs institutional subscription to access the advanced search with full features. I was wondering how you could access it.

Line 25---- Google Scholar isn’t databases rather it’s a search engine. Thus, it’s better to write it separately.

In line 30---- “…...The meta-analysis will be conducted using a random-effects model.” How the authors determine the model before heterogeneity assessment?

Line 32----Why the authors prefer Egger’s test rather than Begg’s test? Or Why not both?

**Background**

The background section is too long. Could the authors make more smart, short and precise background to show the problem for readers in the study area? Most of the ideas are redundant and can be merged. Additionally, the order of paragraphs in this section seems somewhat disorganized (It will be good if the factors come after the prevalence).

Some of the paragraphs are part of methods for example; paragraph 8.

It would be good if the authors include place of the study conducted when they synthesis and compare the previous studies. For example: in line 57 (A recent study in? [10] found…),in line 60, in line 62,…etc.

Line 71----if there is SR and MA of PTSD what will be the contribution/significance of this protocol?

The aim of the study is the prevalence but the authors stated “…understanding how refugees cope with stressors and build resilience can inform impactful mental health interventions and empower individuals”

I can’t find the gap and the significance of the study in the background section.

What is the importance of writing about the ethical issues at the end of the background?

**Objective**

Line 141 objective is not complete.

**Methods**

In your PRISMA figure, I don’t recommend to say “reports excluded” because you are still not sure of getting reports in your search.

Line 148—“The reporting 148 flowchart is illustrated in…” is not complete.

In your inclusion criteria in line 153 spelling error “ault” change to “adult”.

In line 157… you already excluded countries out of East Africa in the title. In addition, It’s not clear why the authors exclude the other East African countries such as Eretria, Djibouti, Madagascar...etc.

Line 160---It’s already known in the title (non-refugees are already excluded in the title)

Line 165---Will you use the two mnemonics (CoCoPop and PEO)? If so how and why?

Line 174---As it’s commented in the abstract section “Google Scholar” is not a database rather a search engine.

**Publication bias**

Line 243---Is there any justification for using the statistical significance p-value 0.10?

Please add risk of bias assessment form

**Discussion**

Web of sciences is one of the databases that you proposed to use in the abstract whereas contradicted in this section.

It will be good, if the authors merged the similar ideas.
