# Supplementary material for: Prevalence and associated factors of depression among adult refugees in East Africa: A protocol for systematic review and meta-analysis
Source: PLoS One. 2025 Feb 11;20(2):e0318904. doi: 10.1371/journal.pone.0318904 (PMC11813089; doi:10.1371/journal.pone.0318904)
Supplement: Attachment — Submitted filename: Comments to the Authors PlosOne.docx [file pone.0318904.s004.docx]

**Comments to the Authors**

The topic “Prevalence and associated factors of depression among adult refugees in East Africa”

is interesting. The manuscript may need to be improved in the following areas:

1) The manuscript repeatedly mentions the study objective, particularly in the Abstract (lines 14-21) and the Introduction (lines 38-50). Concisely restating the objective without repetition will clarify the focus for readers.

2) The introduction mentions the lack of systematic reviews in this area but could further clarify the existing research gap (lines 17-18, 49-50). Expanding on how previous studies are limited such as by geographic scope, lack of consistent diagnostic criteria, or absence of meta-analysis would strengthen the rationale for this review.

3) The inclusion (lines 151-158) and exclusion criteria (lines 159-163) are outlined, but further refinement is suggested. Specifically, explaining the rationale for excluding interventional and qualitative studies would enhance the methodological rigor.

4) The exclusion of EMBASE and Web of Science due to access limitations in Ethiopia (line 252) is a noted limitation, as it may reduce the comprehensiveness of the review. Consider mentioning potential strategies to address this limitation, such as possible collaboration with institutions that have access to these databases.

5) The study emphasis on subgroup analysis is valuable, but more specific examples would add clarity. Identifying potential subgroups, such as geographic regions, gender, or age groups among refugee populations, could enhance the plan’s applicability. The authors briefly address heterogeneity analysis (lines 232-239), but further specifying meta-regression variables for subgroup analysis would justify this methodological choice.

6) The authors intend to use the Joanna Briggs Institute (JBI) checklist for quality assessment (lines 209-216), a commendable approach. However, providing more specific examples for each quality parameter, including definitions for what constitutes high or low quality in this context, would increase transparency. Adding example studies or criteria can enhance the replication potential of this protocol.
