# Supplementary material for: Prevalence and associated factors of depression among adult refugees in East Africa: A protocol for systematic review and meta-analysis
Source: PLoS One. 2025 Feb 11;20(2):e0318904. doi: 10.1371/journal.pone.0318904 (PMC11813089; doi:10.1371/journal.pone.0318904)
Supplement: Attachment — Submitted filename: Response to Reviewers.docx [file pone.0318904.s005.docx]

**Dear reviewer,**

We have special gratitude to you, for devoting your valuable time and energy to review our work entitled “**Prevalence and associated factors of depression among adult refugees in East Africa: A protocol for systematic review and meta-analysis**” for giving constructive comments and valuable guidance. In line with this, the authors had exhaustively demonstrated and addressed questions and comments raised by reviewers using point-by-point responses as stated below.

**Reviewer #1: Comments to the Authors;**
Thank you for the opportunity to review the manuscript titled “Prevalence and associated factors of depression among 1 adult refugees in East Africa: A protocol for systematic review and meta-analysis.”
Overall,
Depression is a significant issue among adult refugees in Africa, as they often face a multitude of stressors and hardships that can contribute to the development of mental health concerns. Refugees are often forced to flee their homes due to violence, persecution, or other traumatic events, which can lead to feelings of helplessness, fear, and loss. However, mental health services for refugees in East Africa are often limited, leading many to suffer in silence without adequate support or treatment.
It is crucial for organizations, governments, and healthcare providers to prioritize mental health support for refugees in Africa, as addressing depression and other mental health concerns can improve their overall well-being and help them to better cope with the challenges they face. Providing access to mental health services, psychosocial support, and community resources can help refugees to heal from their trauma and build resilience as they navigate their new lives.

I hope there are chances to more strengthen this manuscript. Here are the comments to be addressed by the authors.

1. **Abstract**

- Line 19, say the aim of this study is to……

**Response:** Thank you for your suggestion. We incorporated into the revised manuscript.

- write as Materials and Methods through the manuscript

**Response:** Thank you for your suggestion. We incorporated while the revised manuscript.

- Please replace the word reviewers by researchers through manuscript.

**Response:** Thank you for your suggestion. We have replaced the word 'reviewers' with 'researchers' throughout the manuscript as requested.

- Line 32-33 the statement, for determinants of depression an effect size with a 95% confidence interval will be analyzed. Say will be reported.

**Response:** Thank you for your feedback. We have revised the statement in lines 32-33 to read: "For determinants of depression, an effect size with a 95% confidence interval will be reported.

2. **Introduction**

- From Line 49-56 the idea in the paragraph needs the appropriate citation and should be cited?

**Response:** Thank you for your feedback. We have reviewed the content in lines 49-56 and add appropriate citations to ensure the ideas in the paragraph are properly referenced.

- Please cite the idea from Line 103-105?

**Response:** Thank you for your suggestion. We have incorporated it into revised manuscript.

- The paragraph from line 108-115 looks unclear and please modify the paragraph as such in accordance to the rationale for this study or remove it?

**Response:** Thank you for pointing this out. We have removed it to maintain clarity and focus.

- The last paragraph of the introduction looks ambiguous, please modify the last paragraph of the introduction by incorporating the research gaps, and aim of this systematic review and meta-analysis?

**Response:** Thank you for your suggestion. We have incorporated it while revised manuscript.

- Please modify the objective of this study as general and specific forms? Note that your research question should be in line with your specific objective.

**Response:** Thank you for your feedback. We have revised the objectives of the study to include both general and specific forms, ensuring alignment with the research questions.

- Please modify the second specific objective by
  Summarizing associated factors and their effect size among adult refugees in East Africa.

**Response:** Thank you for your feedback. The second specific objective has been revised into revised manuscript.

3. **Materials and Methods**

- Well written.

**Response:** Thank you. we appreciate the feedback.

- Please modify the Materials and Methods section as:
  This systematic review and meta-analysis will be conducted in accordance with the Preferred Reporting Items for Systematic Reviews and Meta Analyses (PRISMA-P) statement. The protocol was registered in the International Prospective Register of Systematic Reviews PROSPERO registration number: CRD42024496728. The PRISMA flow chart for reporting systematic review and meta-analysis is presented in figure 1.

**Response:** Thank you for the detailed suggestion. We have incorporated the modifications as outlined, ensuring the Materials and Methods section aligns with the PRISMA-P statement and includes the specified registration details and flow chart reference. Let us know if there is anything else you would like adjusted.

- In the eligibility criteria, the best way to organize the search terms for the study is the CoCoPop criteria (condition, context, and population). However, the authors should mention these criteria and modify the inclusion criteria.

**Response:** Thank you for the insightful suggestion. We have ensured the CoCoPop framework is mentioned in the eligibility criteria and have revised the inclusion criteria accordingly for better alignment. we appreciate your guidance.

- Please merge the CoCoPop criteria in the inclusion criteria

**Response:** Thank you for the clarification. We have ensured the “search guide” and “eligibility criteria” are treated as a separate section. Separating the search guide and eligibility criteria into two sections ensures clarity and organization. The search guide can focus on the systematic approach to study identification, while the eligibility criteria define inclusion and exclusion standards. This separation improves ease of reference, supports thorough documentation, and simplifies review and revision processes, leading to a more transparent and structured systematic review. Please let me know if there are any additional details or formatting preferences to consider.

- Next to the eligibility criteria please add the subtitle Outcome measures and describe the outcomes of this systematic review and meta-analysis in detail?

**Response:** Thank you for the suggestion. We have added the 'Outcome Measures' subtitle next to the eligibility criteria and provide a detailed description of the outcomes for the systematic review and meta-analysis.

- In the search strategy please provide the full proposed search strategy for all proposed databases?

**Response:** Thank you for the suggestion. We have included the full proposed search strategies for other databases in the **supplementary files** as requested.

- Subgroup analyses and meta-regressions will be performed to examine sources of heterogeneity. In relation to this study what are the proposed variables for Subgroup analyses and meta-regressions, please mention?

**Response:** Thank you for the question. For this study, the proposed variables for subgroup analyses and meta-regressions include sex (female vs. male), social support (poor social support vs. good social support), and cumulative traumatic events (≥8 vs. <8). These variables will be investigated to examine potential sources of heterogeneity and assess the robustness of the findings in relation to the pooled prevalence of depression and associated factors. We have mentioned while revised the protocol.

4. **Discussion**

- Please modify the discussion part as such, containing detailed description of the research question and objectives, the justification for conducting the review, a summary of the existing literature on the topic, and an explanation of the methods that will be used to select and analyze studies for inclusion in the review. Moreover, the discussion section may also discuss any potential limitations or biases in the review process, as well as how the findings of the review will be interpreted and disseminated?

**Response:** Thank you for the detailed guidance. We have revised the discussion section to include a more comprehensive description of the research question, objectives, justification for the review, a summary of the literature, and a clear explanation of the methods. We have also addressed potential limitations and how the findings will be interpreted and disseminated

- Modify the strength an and limitation, for instance,
  The statements about publication bias will be performed by two authors is not the strength, Also the absence of meta-analysis study is not the limitation of this study, please modify accordingly?

**Response:** Thank you for the helpful feedback. We have revised the strengths and limitations section as requested, ensuring the points about publication bias and the absence of meta-analysis studies are adjusted accordingly.

5. **References**

- Well written

**Response:** Thank you. we appreciate the feedback.

- Make sure that all cited sources are in their appropriate and supporting ideas.

**Response:** Thank you for your suggestion. We have incorporated it into revised manuscript.

**General Comments**

- The authors should modify the format of this protocol according to the PLOS ONE guideline i.e INTRODUCTION, MATERIALS AND METHODS and DISCUSSION.

**Response:** Thank you for the important suggestion. We have revised the format of the protocol to align with the PLOS ONE guidelines, restructuring it into the specified sections: INTRODUCTION, MATERIALS AND METHODS, and DISCUSSION

- The author should check typo and grammars order to increase the quality of the manuscript.

**Response:** Thank you for your suggestion. We have incorporated into revised protocol.

**Dear reviewer,**

We have special gratitude to you, for devoting your valuable time and energy to review our work entitled “**Prevalence and associated factors of depression among adult refugees in East Africa: A protocol for systematic review and meta-analysis**” for giving constructive comments and valuable guidance. In line with this, the authors had exhaustively demonstrated and addressed questions and comments raised by reviewers using point-by-point responses as stated below.

**Reviewer #2:** Title: Prevalence and associated factors of depression among adult refugees in East Africa: A protocol for systematic review and meta-analysis.
I would like to thank you the editor to give me the opportunity to review this interested protocol which focused on the unusual/refugee settings. I hope the review protocol will help to design strategies for the improvement of the health of refugees particularly in the settings with natural and man-made catastrophes. Here below are some of my comments.
**Abstract**

- Line 23--- I think web of sciences needs institutional subscription to access the advanced search with full features. I was wondering how you could access it.

**Response:** You are absolutely right. Accessing **Web of Sciences** advanced search features does require an institutional subscription. Since we do not have access, we will focus on other databases for our search strategy. So, we have removed it while revised protocol.

- Line 25---- Google Scholar isn’t databases rather it’s a search engine. Thus, it’s better to write it separately.

**Response:** Thank you for pointing that out. We have revised the sentence to separate Google Scholar from the databases and clarify its role as a search engine into revised protocol.

- In line 30---- “…...The meta-analysis will be conducted using a random-effects model.” How the authors determine the model before heterogeneity assessment?

**Response:** Thank you for raising this important point. We might be pre-selecting a random-effects model based on the expectation of variability among the included studies, such as differences in study populations, methodologies, or outcomes. This approach assumes that the true effects may vary between studies, making a random-effects model suitable for pooling results. However, the final decision on the model will be guided by the heterogeneity assessment, typically using measures such as the I² statistic or Cochran's Q test. If the heterogeneity is minimal, a fixed-effect model may be considered as an alternative.

- Line 32----Why the authors prefer Egger’s test rather than Begg’s test? Or Why not both?

**Response:** We prefer Egger’s test over Begg’s test because Egger’s test is generally considered more sensitive for detecting small-study effects and potential publication bias, particularly in meta-analyses with a smaller number of studies. While Begg’s test is less prone to type I errors (false positives), it may lack power compared to Egger’s test in certain scenarios. That said, the choice between these tests depends on the specific context of the meta-analysis. In some cases, using both tests can provide complementary insights: Egger’s test for sensitivity and Begg’s test for confirmation. If deemed appropriate, we may include both tests to provide a robust assessment of publication bias.

**Background**

- The background section is too long. Could the authors make more smart, short and precise background to show the problem for readers in the study area? Most of the ideas are redundant and can be merged. Additionally, the order of paragraphs in this section seems somewhat disorganized (It will be good if the factors come after the prevalence).
  Some of the paragraphs are part of methods for example; paragraph 8. It would be good if the authors include place of the study conducted when they synthesis and compare the previous studies. For example: in line 57 (A recent study in? [10] found…), in line 60, in line 62,…etc.

**Response:** Thank you for your valuable feedback. We appreciate your suggestion to condense the background section to make it more concise and focused. We have revised the section to ensure it is more streamlined by merging redundant ideas and reordering the content so that the prevalence is presented before the factors.

- Line 71----if there is SR and MA of PTSD what will be the contribution/significance of this protocol? The aim of the study is the prevalence but the authors stated “…understanding how refugees cope with stressors and build resilience can inform impactful mental health interventions and empower individuals” I can’t find the gap and the significance of the study in the background section. What is the importance of writing about the ethical issues at the end of the background?

**Response:** Thank you for your insightful comments. We understand your concern regarding the contribution and significance of this protocol, especially considering the existing systematic reviews (SR) and meta-analyses (MA) on PTSD. While there is existing literature on PTSD among refugees, this study specifically focuses on depression, a critical but often underexplored mental health issue within this population. By concentrating on the prevalence of depression and its associated risk factors in East African refugees, our protocol aims to fill a gap in understanding the broader mental health landscape, distinct from PTSD, which can lead to targeted interventions. The significance of this study lies in its ability to provide robust, region-specific data on depression, which is crucial for developing culturally relevant mental health policies and interventions tailored to refugees in East Africa. We appreciate your suggestions and have revised the background section to better emphasize the study’s unique contribution and significance while improving the overall structure.

**Objective**

- Line 141 objective is not complete.

**Response:** Thank you for pointing that out. We have revised it to ensure it fully conveys the scope and purpose of the study into revised protocol.

**Methods**

- In your PRISMA figure, I don’t recommend to say “reports excluded” because you are still not sure of getting reports in your search.

**Response:** Thank you for your suggestion. W e have incorporated into revised protocol.

- Line 148—“The reporting 148 flowchart is illustrated in…” is not complete.

**Response:** Thank you for your suggestion. We have completed the sentence while the revised protocol.

- In your inclusion criteria in line 153 spelling error “ault” change to “adult”.

**Response:** Thank you for noticing the typographical error in the inclusion criteria. We have promptly correct "ault" to "adult" in line 153 to ensure accuracy and clarity in the text. We appreciate your careful review and attention to detail.

- In line 157… you already excluded countries out of East Africa in the title. In addition, It’s not clear why the authors exclude the other East African countries such as Eretria, Djibouti, Madagascar...etc.

**Response:** Thank you for your observation. To clarify, we will not exclude any East African countries in our inclusion criteria. The study encompasses all countries within the East African region, including Eritrea, Djibouti, and Madagascar, provided relevant studies addressing the prevalence and risk factors of depression among adult refugees are available. We will revise line 157 to ensure this inclusivity is clearly stated and consistent with the scope of the study described in the title. We appreciate your feedback and will make the necessary corrections for greater clarity.

- Line 160---It’s already known in the title (non-refugees are already excluded in the title)

**Response:** Thank you for your feedback. We agree that the exclusion of non-refugee populations is already implicit in the title, and we have revised the text to avoid redundancy into revised protocol.

- Line 165---Will you use the two mnemonics (CoCoPop and PEO)? If so how and why?

**Response:** Thank you for your thoughtful question. In this study, we will primarily employ the CoCoPop framework (Condition, Context, and Population) to design our search strategy. The CoCoPop framework is particularly well-suited for this systematic review and meta-analysis as it aligns directly with our study objectives by focusing on the prevalence of depression (Condition), adult refugees (Population), and the East African region (Context).

While the PEO (Population, Exposure, Outcome) mnemonic is another valuable tool, it is typically more appropriate for studies examining exposures and their direct outcomes. Since our study is primarily descriptive and aims to estimate prevalence and identify associated factors rather than investigating specific exposures, CoCoPop provides a more targeted and efficient framework. So, we have removed the PEO while revised protocol.

- Line 174---As it’s commented in the abstract section “Google Scholar” is not a database rather a search engine.

**Response:** Thank you for your suggestion. We have incorporated it while revised protocol.

**Publication bias**

- Line 243---Is there any justification for using the statistical significance p-value 0.10?

**Response:** Thank you for your observation. Apologies for this mistake. In this study, we have chosen a p-value threshold of <0.05 to determine statistical significance, as it is a widely accepted standard in many fields of research, including systematic reviews and meta-analyses. This threshold ensures a balance between Type I and Type II error rates, providing robust evidence for statistical findings. Using a p-value of <0.10 might be considered in exploratory analyses or in cases where a more lenient threshold is warranted due to limited sample sizes or high variability. However, given the scope and objectives of our study, we opted for the more stringent <0.05 threshold to ensure the reliability and validity of our findings. We have incorporated it while revised protocol.

- Please add risk of bias assessment form

**Response:** Thank you for your suggestion. We have included a risk of bias assessment form in the protocol to ensure a thorough evaluation of the methodological quality of the included studies.

**Discussion**

- Web of sciences is one of the databases that you proposed to use in the abstract whereas contradicted in this section. It will be good, if the authors merged the similar ideas.

**Response:** Thank you for pointing that out. We apologize for the inconsistency between the abstract and the methods section regarding the inclusion of the Web of Science database. Upon review, we have decided not to include Web of Science due to access limitations. We have revised both sections to ensure consistency and merge similar ideas to provide a clear and accurate representation of the search strategy. Thank you for bringing this to our attention, and we have made the necessary adjustments.

**Dear reviewer,**

We have special gratitude to you, for devoting your valuable time and energy to review our work entitled “**Prevalence and associated factors of depression among adult refugees in East Africa: A protocol for systematic review and meta-analysis**” for giving constructive comments and valuable guidance. In line with this, the authors had exhaustively demonstrated and addressed questions and comments raised by reviewers using point-by-point responses as stated below.

**Reviewer #3: Comments to the Authors**
The topic “Prevalence and associated factors of depression among adult refugees in East Africa”
is interesting. The manuscript may need to be improved in the following areas:

1) The manuscript repeatedly mentions the study objective, particularly in the Abstract (lines 14-21) and the Introduction (lines 38-50). Concisely restating the objective without repetition will clarify the focus for readers.

**Response:** Thank you for your feedback. We have revised the protocol to avoid repetition of the study objective. The objective was concisely stated in the abstract and introduction, ensuring clarity without redundancy.

2) The introduction mentions the lack of systematic reviews in this area but could further clarify the existing research gap (lines 17-18, 49-50). Expanding on how previous studies are limited such as by geographic scope, lack of consistent diagnostic criteria, or absence of meta-analysis would strengthen the rationale for this review.

**Response:** Thank you for your suggestion. We have revised the introduction to more clearly articulate the research gap by highlighting the limitations of existing studies, such as geographic scope, inconsistent diagnostic criteria, and the lack of meta-analysis.

3) The inclusion (lines 151-158) and exclusion criteria (lines 159-163) are outlined, but further refinement is suggested. Specifically, explaining the rationale for excluding interventional and qualitative studies would enhance the methodological rigor.

**Response:** Thank you for the suggestion. We have clarified the rationale for excluding interventional and qualitative studies. Interventional studies are excluded because they focus on evaluating specific interventions, which does not align with the aim of this review, which is to estimate the prevalence of depression and identify associated factors. Qualitative studies are excluded as they do not provide the quantitative data necessary for meta-analysis, which is central to the synthesis of pooled estimates in this review. We have updated the protocol to include this explanation for better methodological rigor.

4) The exclusion of EMBASE and Web of Science due to access limitations in Ethiopia (line 252) is a noted limitation, as it may reduce the comprehensiveness of the review. Consider mentioning potential strategies to address this limitation, such as possible collaboration with institutions that have access to these databases.

**Response:** Thank you for your feedback. While we acknowledge that the exclusion of EMBASE and Web of Science due to access limitations in Ethiopia may reduce the comprehensiveness of the review, we have decided to proceed with the current inclusion of other databases that provide sufficient coverage of the topic. At this stage, we do not intend to explore collaboration with other institutions for accessing these databases. We believe the chosen databases (PubMed, CINAHL, AJOL, and Google Scholar) provide a comprehensive representation of the available literature for this review. Therefore, we have not planned any specific strategies to address this limitation at present.

5) The study emphasis on subgroup analysis is valuable, but more specific examples would add clarity. Identifying potential subgroups, such as geographic regions, gender, or age groups among refugee populations, could enhance the plan’s applicability. The authors briefly address heterogeneity analysis (lines 232-239), but further specifying meta-regression variables for subgroup analysis would justify this methodological choice.

**Response:** Thank you for your valuable suggestion. We appreciate your emphasis on specifying subgroups for the analysis. To enhance the clarity of the plan, we have provided more detailed examples of potential subgroups for subgroup analysis and meta-regressions.

6) The authors intend to use the Joanna Briggs Institute (JBI) checklist for quality assessment (lines 209-216), a commendable approach. However, providing more specific examples for each quality parameter, including definitions for what constitutes high or low quality in this context, would increase transparency. Adding example studies or criteria can enhance the replication potential of this protocol.

**Response:** Thank you for your valuable suggestion. We agree that providing further clarification on the quality assessment parameters will enhance transparency and the replicability of this protocol. We have incorporated it while revised protocol.
