# Supplementary material for: Prevalence and associated factors of depression among adult refugees in East Africa: A protocol for systematic review and meta-analysis
Source: PLoS One. 2025 Feb 11;20(2):e0318904. doi: 10.1371/journal.pone.0318904 (PMC11813089; doi:10.1371/journal.pone.0318904)
Supplement: Attachment — Submitted filename: Response to Reviewers.docx [file pone.0318904.s006.docx]

**Dear Editor,**

We have special gratitude to you, for devoting your valuable time and energy to review our work entitled “**Prevalence and associated factors of depression among adult refugees in East Africa: A protocol for systematic review and meta-analysis**” for giving constructive comments and valuable guidance. In line with this, the authors had exhaustively demonstrated and addressed suggestions and comments raised by editor using point-by-point responses as stated below.

**Additional Editor Comments:**
Thank you so much for your revision, which you have well addressed to our reviewers suggestions. After carefully reviewing your revised manuscript, I would suggest addressing the following before final approval for the publication in this journal:
a) Could you please keep only review questions and remove lines 85–91 (general objectives and specific objectives), which appear repetitive?

**Response:** **Thank you for bringing this to our attention. We have removed lines 85–91, which contained the general and specific objectives, as they appeared repetitive. Only the review questions have been retained as requested. Please let us know if there’s anything else you would like us to adjust.**
b) Could you edit and make corrections to line 100? Make a section heading: Inclusion and exclusion criteria, and explain your inclusion criteria, i.e., data include from inception to include the month that is missing in line 102, i.e., July 2024?

**Response:** **Thank you for the feedback. We have edited and corrected line 100 as requested. A section heading, 'Inclusion and exclusion criteria,' has been added, and the inclusion criteria have been updated to specify that data includes studies published up to July 2024 in the revised manuscript. Please let us know if there are any additional adjustments you would like.**
c) Make clear in lines 106-108 how many countries are in East Africa and accordingly state all countries names in alphabetical order.

**Response: Thank you for the suggestion. Lines 106–108 have been updated to specify that there are 18 countries in East Africa, now listed in alphabetical order: Burundi, Djibouti, Eritrea, Ethiopia, Kenya, Madagascar, Rwanda, Somalia, South Sudan, Tanzania, and Uganda in the revised manuscript. Please let us know if further changes are needed**
d) Lines between 117 and 121 need more clarity, particularly on the “CoCoPop search guide,” which search frameworks need citation to back up this terminology where you can see several other frameworks, i.e., PICO, PCC, PEO, SPICE, SPIDER, and widely used in several systematic review works. So, make this very clear by providing the citation to back up your statement or adopting the widely used framework.

**Response: Thank you for your valuable feedback. We have revised lines 117–121 to provide greater clarity on the 'CoCoPop search guide’ in the revised manuscript. Citations have been added to substantiate the use of this framework.**
e) In lines 122 to 126, clarify that depression-related factors are secondary results, despite the title implying that both prevalence and associated factors are primary outcomes of this review.

**Response:** **Thank you for your feedback. We have revised lines 122–126 to clarify that while the title might suggest both prevalence and associated factors are primary outcomes, the depression-related factors are actually secondary outcomes.** **We have clarified in the revised manuscript as 'The purpose of this review is to assess two outcomes: the first is to determine the pooled prevalence of depression among adult refugees in East Africa, and the second is to identify the factors associated with depression’.**
f) Line 127: Keep only the Medline database, which adequately covers PubMed; there is no need to include PubMed with Medline.
**Response:** **Thank you for your suggestion. We have revised line 127 to retain only the Medline database, as it sufficiently covers PubMed. The reference to PubMed has been removed to streamline the information both in the Abstract and the body of the text. Please let us know if any further adjustments are needed.**
g) In line 129, Google Scholar is mentioned as a search resource, which is untrustworthy for systematic review and meta-analysis searches. Replace Google Scholar with the PsycINFO database, which is crucial to your psychological research topic.

**Response:** **Thank you for your valuable feedback. We have updated line 129 to replace Google Scholar with the PsycINFO database, which is more reliable for systematic review and meta-analysis searches, especially for psychological research in the revised manuscript. Please let us know if there are any other adjustments you would like to make.**
h) I would also recommend you keep your search terminologies (Table 1) in the appendix.

**Response:** **Thank you for your recommendation. We have incorporated this revision into the manuscript and included the terminologies in the supporting information as 'S1'.**
