# Supplementary material for: Prevalence and associated factors of depression among adult refugees in East Africa: A protocol for systematic review and meta-analysis
Source: PLoS One. 2025 Feb 11;20(2):e0318904. doi: 10.1371/journal.pone.0318904 (PMC11813089; doi:10.1371/journal.pone.0318904)
Supplement: Attachment — Submitted filename: Response to Reviewers.docx [file pone.0318904.s007.docx]

**Dear Editor,**

We have special gratitude to you, for devoting your valuable time and energy to review our work entitled “**Prevalence and associated factors of depression among adult refugees in East Africa: A protocol for systematic review and meta-analysis**” for giving constructive comments and valuable guidance. In line with this, the authors had exhaustively demonstrated and addressed suggestions and comments raised by editor using point-by-point responses as stated below.

 -Thoroughly review the report, i.e., lines 66 and 91, and edit and maintain the consistency, i.e., line 135 (AHS&AAS) and line 143 (AHS and AAS). So, use "and" not "&" in academic writing.

**Response: Thank you for the feedback. We have edited and corrected while in to the revised manuscript.**
- Line 68 defines first-time use of abbreviation "LGBTQ+"

**Response: Thank you for the suggestion. We have included while into revised manuscript.**
- Not sure what you intend to state in line 390 is, so thoroughly proofread,

**Response: Thank you for the suggestion. We have removed it and can assessed from supporting information material.**
- 26-31 citations are inconsistent and avoid repetition, i.e., 28 and 31,

**Response: Thank you for the suggestion. We have removed the repeated reference.**
- Make correction of line 190 after 39 reference full stops,

**Response: Thank you for the suggestion. We have incorporated while revised the manuscript.**
- Still not corrected and stated the consistency of the use of databases, i.e., line 219 Google Scholar,

**Response: Thank you very much for your insightful suggestion. We have incorporated while revised into the manuscript.**
- Avoid multiple times defining abbreviations, i.e., line 27, 144, 239 Joanna Briggs Institute (JBI).

**Response: Thank you for your suggestion. We have incorporated into the revised manuscript.**
